# A Case Report of Central Bronchiectasis in a Poorly Controlled Asthmatic Adolescent With Allergic Bronchopulmonary Aspergillosis and Secondary Spontaneous Pneumothorax

**DOI:** 10.7759/cureus.64792

**Published:** 2024-07-18

**Authors:** Kasireddy Sravanthi, Devika Jadhav, Sampada Tambolkar, Shailesh B Meshram, Manojkumar G Patil, Shailaja Mane

**Affiliations:** 1 Pediatrics, Dr. D. Y. Patil Medical College, Hospital & Research Centre, Dr. D.Y. Patil Vidyapeeth, Pune, IND; 2 Respiratory Medicine, Dr. D. Y. Patil Medical College, Hospital & Research Centre, Dr. D.Y. Patil Vidyapeeth, Pune, IND

**Keywords:** poorly controlled asthma, bronchiectasis, eosinophilia, asthma, allergic bronchopulmonary aspergillosis

## Abstract

Allergic bronchopulmonary aspergillosis (ABPA) is a multifaceted immune hypersensitivity reaction occurring in the lungs and bronchi, triggered by exposure and colonization of *Aspergillus* species, commonly *Aspergillus fumigatus (A. fumigatus)*. It typically affects individuals who are immunocompetent but predisposed, such as those with bronchial asthma and cystic fibrosis. Diagnosis involves various methods including chest radiography, computed tomography, identification of eosinophilia, elevated serum IgE (immunoglobulin E) levels, and immunological tests for *Aspergillus *antigen. Left undiagnosed and untreated, ABPA can advance to bronchiectasis and/or pulmonary fibrosis, leading to significant morbidity and mortality.

## Introduction

*Aspergilli*, a widespread type of fungi, consist of approximately 185 species, with *A. fumigatus, Aspergillus niger*, and *Aspergillus flavus* being the most prevalent ones known to cause diseases in humans. Among these, *A. fumigatus *predominantly contributes to cases of ABPA, a condition characterized by an inflammatory reaction to *Aspergillus* present in the mucus of individuals with underlying lung conditions such as persistent asthma and cystic fibrosis (CF). Various immune factors, such as atopy and specific immunogenic human leukocyte antigen (HLA) types in asthma patients, as well as genetic factors like mutations in the CF transmembrane conductance regulator (CFTR) gene in CF patients, elevate the susceptibility to ABPA in these populations [1].

ABPA manifests in around 1-2% of children with asthma and 2-15% of individuals with CF [2]. Establishing the prevalence of ABPA in asthmatics has proven problematic due to the absence of standardized diagnostic criteria. Consider ABPA in patients with chronic breathing difficulties (airway limitation) and a pre-existing lung condition like asthma or CF. A positive skin test result for *Aspergillus*, increased serum IgE (>417 IU/L), fungal-specific IgE and IgG antibodies, and imaging modalities are all part of the diagnostic workup for ABPA [1].

## Case presentation

A 13-year-old male child presented to the outpatient department with complaints of fever, cough, and worsening asthma symptoms such as dyspnea and episodic wheezing for three years. His cough was productive, yellowish, and non-blood-stained. He was admitted with similar complaints in the past four times and was treated for pneumonia. His symptoms were relieved with treatment but didn’t improve completely. He was diagnosed with asthma at the age of eight years and progressed gradually over the past three years. He was managed with inhaled corticosteroids and short-acting beta-agonists. Spirometry showed significant bronchodilator reversibility of +23.23% and 240 ml.

**Figure 1:**
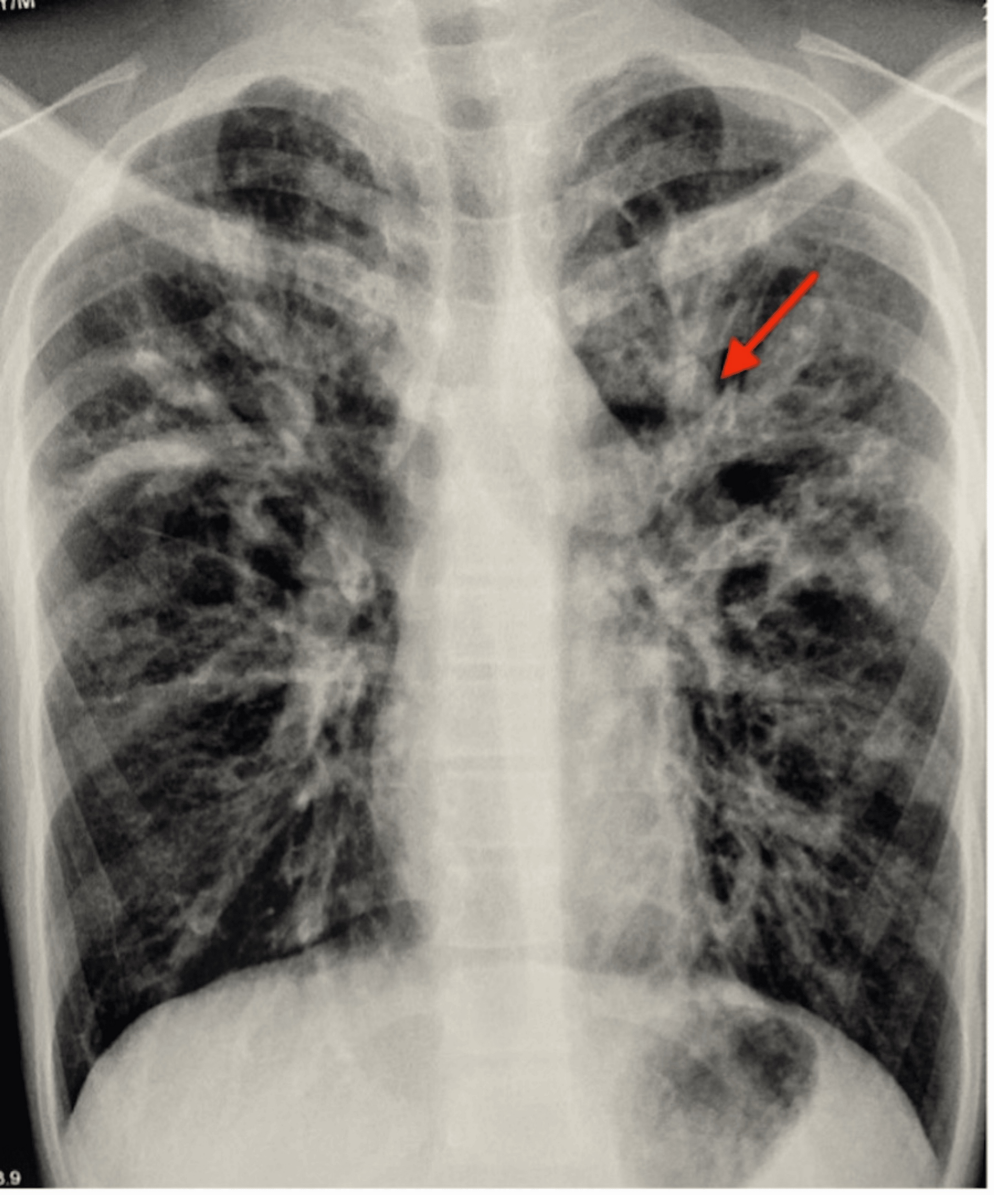
Chest X-ray showing gloved finger appearance Red arrow: gloved finger appearence

On admission, the child was afebrile with a pulse of 102 beats per minute, respiration rate of 38 per minute, peripheral pulses well felt, oxygen saturation of 84% on room air and blood pressure of 100/72 mm Hg. Saturation improved to 91% on oxygen by mask. On general examination, mild cyanosis and clubbing were present. Auscultation of the chest revealed bilaterally equal air entry and bilateral crepitations with expiratory wheeze. The child was conscious and alert. Further physical examination demonstrated no other pathological findings. Family history was not significant. Routine laboratory investigations and chest X-rays were done as shown in Table 1 and Figure 1.

**Table 1:** Routine laboratory investigations TLC: total leucocyte count; RBC: red blood cell; CRP: C-reactive protein

Parameter (Reference Range)	Lab Value
Hemoglobin (12.8-16 g/dL)	11
TLC (4000-9100/µL)	11,700
Neutrophils (40-70%)	60%
Eosinophils (1-6%)	9%
Lymphocytes (20-40%)	23%
Platelet count (150000-410000/µL)	3,55,000
Packed Cell Volume (37.3-47.7%)	35.4
RBC count (4.4-5.5 million/µL)	5.08
CRP (<2mg/L)	11.1
Erythrocyte sedimentation rate (Up to 15mm/hr)	25
Alanine aminotransferase (7-55 U/Lt)	8
Aspartate aminotransferase (8-60U/Lt)	12
Creatinine (0.35-0.86 mg/dl)	0.63

The child having experienced multiple episodes of pneumonia following asthma treatment combined with the clinical picture, tuberculosis infection was suspected. Given India's status as a developing nation with tuberculosis prevalence, a thorough tuberculosis workup was conducted, but laboratory and radiological findings ruled out the infection. A sweat chloride test was done to rule out CF which returned to be negative. Despite this, clinical and laboratory assessments pointed towards uncontrolled asthma as the likely diagnosis.

**Figure 2:**
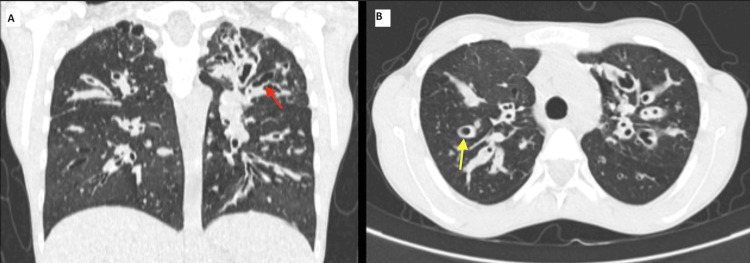
A computed tomography chest showing tubular and cystic bronchiectasis Red arrow: tubular bronchiectasis; yellow arrow: cystic bronchiectasis

A high-resolution computed tomography was done, which revealed extensive areas of cystic, tubular and varicoid bronchiectasis with bronchial wall thickening of the subsegmental and segmental bronchi giving the finger-in-glove appearance in all the lobes of bilateral lungs with central distribution suggestive of ABPA, as shown in Figure 2.

Additionally, blood tests indicated peripheral eosinophilia with an absolute eosinophil count of 1053/ µL and significantly elevated serum total IgE 2428 IU/ml, positive blood test for *Aspergillus* specific IgE 49.6kUA/L and *Aspergillus* specific IgG 142 mgA/L, as listed in Table 2. His blood culture was negative, but sputum culture tested positive for *Pseudomonas aeruginosa.* 

**Table 2:** Further laboratory investigations IgE: Immunoglobulin E; IgG: immunoglobulin G

Parameter (Reference Range)	Lab Value
Absolute Eosinophil count (0-500/ µL)	1053
Serum total IgE (100-200 IU/ml)	2428
*Aspergillus* specific IgE (<0.1 kUA/L)	49.6
*Aspergillus* specific IgG (>30 mgA/L)	142

**Figure 3:**
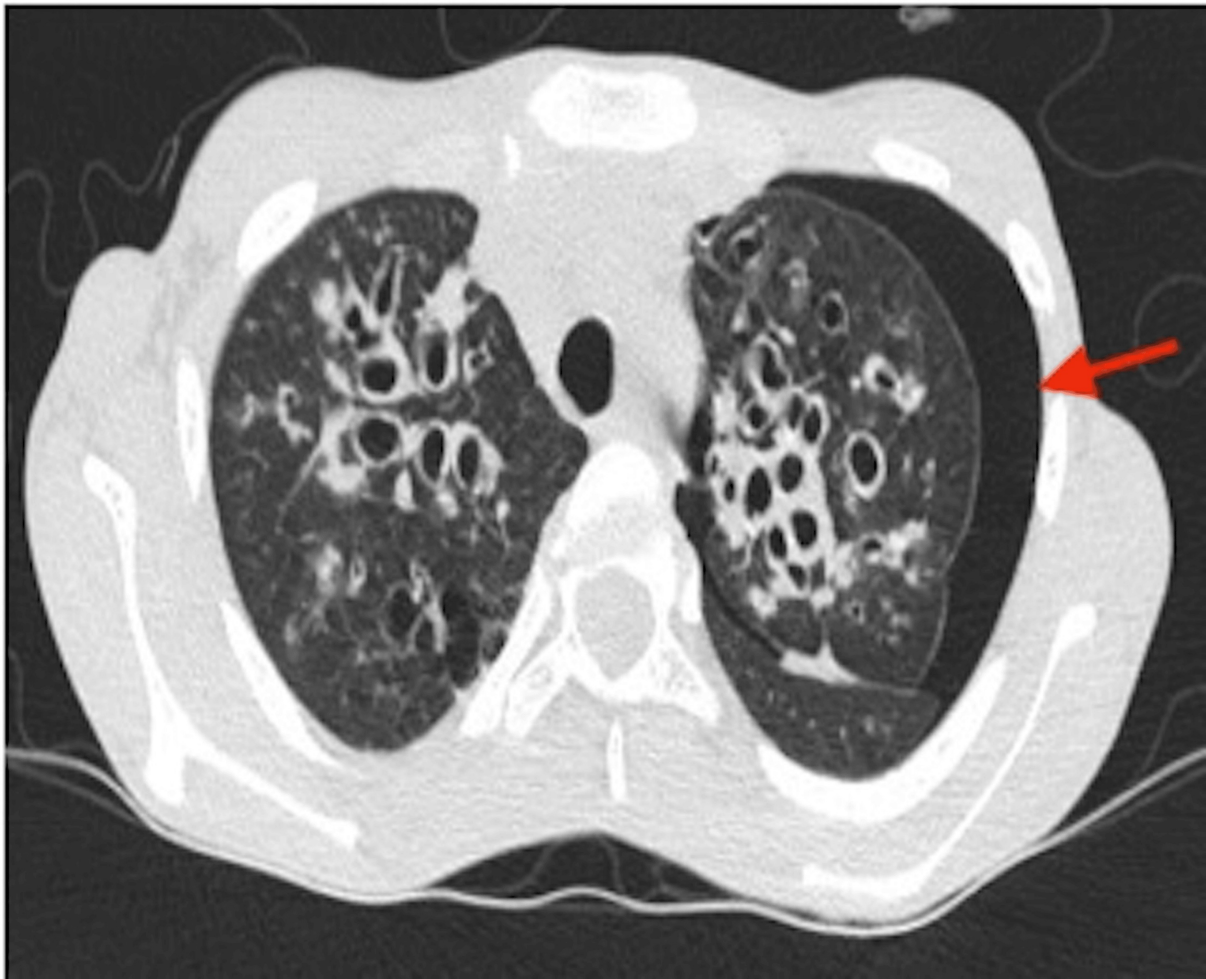
CT showing left-sided pneumothorax Red arrow: pneumothorax

**Figure 4:**
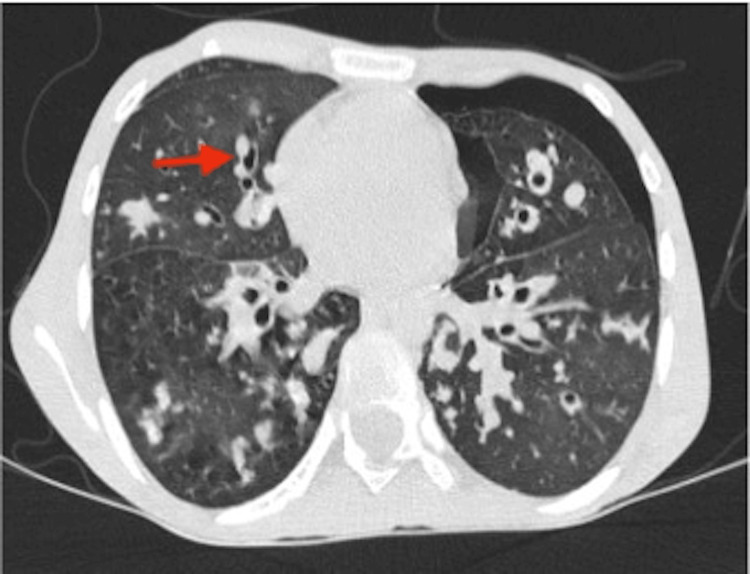
CT image showing signet ring sign. Note that the dilated airway diameter is greater than the diameter of the adjacent blood vessel Red arrow: signet ring sign

**Figure 5:**
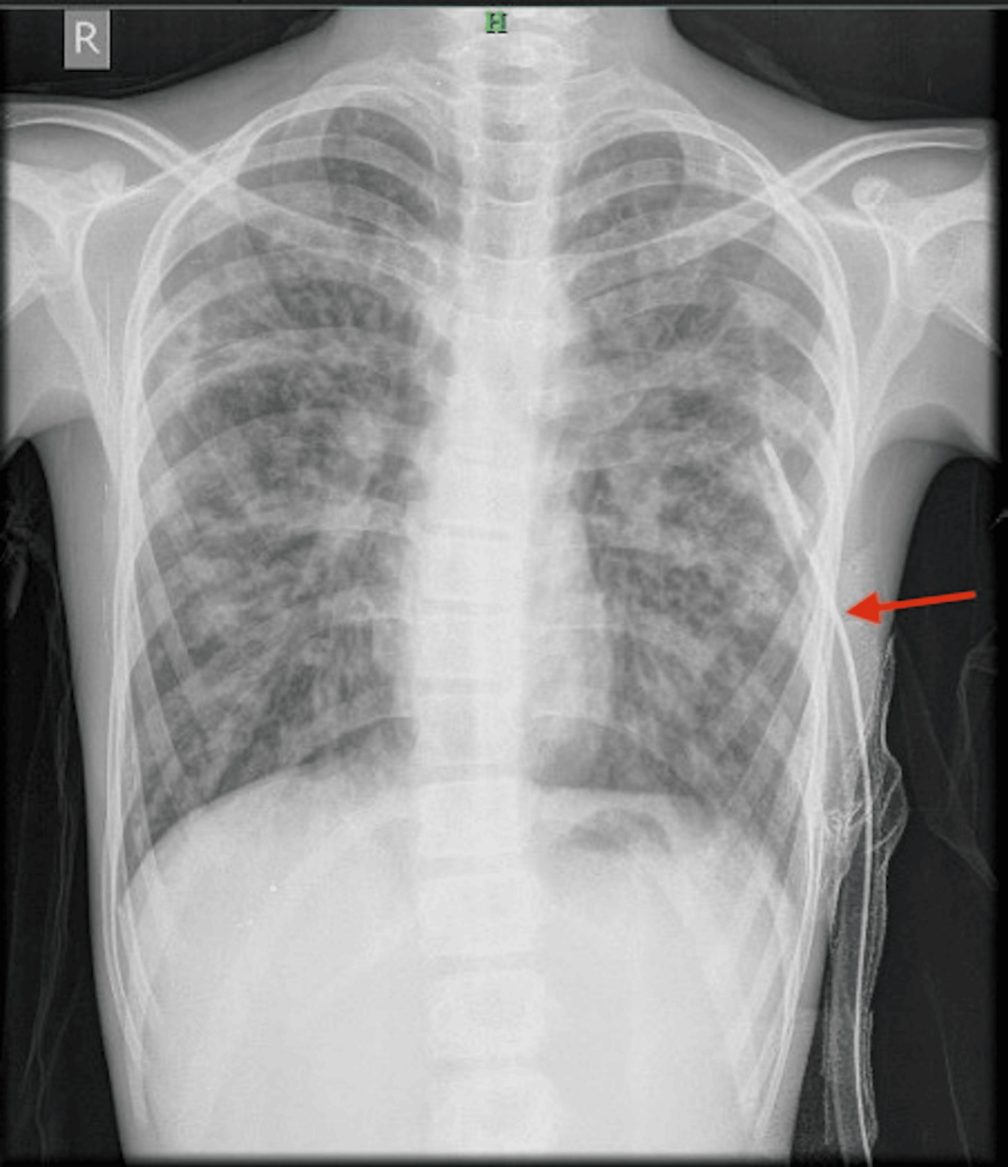
Chest X-ray with intercostal tube in situ Red arrow: intercostal tube

Despite proper management, the child did not improve. On day-3 of admission, repeat chest radiography and computed tomography (CT) were done in view of no clinical improvement, which showed left-sided moderate pneumothorax as shown in Figure 3, and a characteristic signet ring sign was seen as shown in Figure 4. Pneumothorax was managed by inserting an intercostal tube as shown in Figure 5. The child's condition improved significantly, and after confirming lung re-expansion on chest X-ray, the chest tube was safely removed after seven days. After stabilizing the patient, a bronchoscopy procedure was performed revealing mucus plugs in the airways, which were cleared and sent for investigation. Bronchoalveolar lavage (BAL) for malignant cytology and CBNAAT (Cartridge-Based Nucleic Acid Amplification Test) tested negative. KOH mount of BAL showed branched septate hyphae and spores. This finding further supported the diagnosis of ABPA.

The child was given Prednisolone at 0.5mg/kg/day for two weeks followed by 0.5mg/kg on alternate days for the next four weeks, slowly tapered and stopped over four months. Itraconazole 200mg/day was given for 16 weeks. After regular evaluations (every month), the exacerbations became less frequent, and the need for follow-up was reduced. He showed significant improvement in his health and consistently managed his daily routine after six months.

## Discussion

ABPA is characterized by a complex interplay of immune responses to *A. fumigatus.* Although the precise pathophysiology is yet unknown, a number of immunological responses are seen, such as abnormal T-lymphocyte responses and A. fumigatus-specific IgE-mediated type-1 and type-3 hypersensitivity. Upon inhalation, *A. fumigatus *spores germinate into hyphae within the bronchi, releasing diverse proteins and toxins that trigger inflammatory responses. Individuals with asthma and CF who develop ABPA often exhibit genetic susceptibility factors. For instance, the absence of HLA-DQ2 and the presence of HLA-DR2 and/or HLA-DR5 increase the risk of ABPA upon exposure to *Aspergillus*. Conversely, the presence of HLA-DQ2 is believed to confer a protective effect against ABPA [3,4].

The diagnosis of ABPA is a challenging task because of the overlap in both clinical and radiographic symptoms. A mix of clinical examination, laboratory studies, and radiographic results is used to make the diagnosis, as shown in Tables 3, 4.

**Table 3:** Diagnostic criteria for ABPA in asthma * Inner 2/3rd of chest CT field; AF: *Aspergillus fumigatus*; ABPA: allergic bronchopulmonary aspergillosis; IgE: immunoglobulin E; IgG: immunoglobulin G

Criteria	ABPA Central Bronchiectasis	ABPA-Seropositive
Essential Criteria	Asthma Central bronchiectasis* Immediate skin sensitivity to *Aspergillus* species or AF	Asthma Immediate skin sensitivity to* Aspergillus *species or AF Total serum IgE conc. > 417 kU/L (1000 ng/mL) Elevated serum IgE and/or IgG-AF
Non-essential Criteria	Chest X-ray infiltrates Serum precipitating antibodies to AF	Chest X-ray infiltrates

**Table 4:** Changing diagnostic criteria for ABPA in asthma with time AF: *Aspergillus fumigatus*, Total IgE (Immunoglobulin E): 1 kU/L=2.4 ng/mL, 1 kU/L=1 IU/ml, *Transient (nodules, consolidation, tram‑track sign, fleeting opacities, finger in glove/toothpaste opacities) or fixed (ring shadows, bronchiectasis, or fibrosis); ABPA: allergic bronchopulmonary aspergillosis, CF: cystic fibrosis, CT: computerized tomography

Rosenberg‑Patterson Criteria 1977 [5]	Greenberger Criteria 2002 [6]		Agarwal et al. 2013 [7]	Agarwal et al. 2016 [8]
ABPA very likely if the first 6 of 7 primary fulfilled. ABPA certain if all primary 7 present	ABPA‑central bronchiectasis	ABPA‑seropositive	ABPA is diagnosed if all of following criteria are met	ABPA is diagnosed if all of the following criteria are met
Primary	Essential criteria	Essential criteria		
1. Asthma	1. Asthma	1. Asthma	1. Predisposing condition‑Asthma or cystic fibrosis	1. Predisposing condition‑Asthma or cystic fibrosis, COPD, post‑TB fibrocavitary disease
2. Peripheral blood eosinophilia (>1.0×109/L)	2. Immediate skin sensitivity to *Aspergillus* species or AF	2. Immediate skin sensitivity to *Aspergillus* species or AF	2. Obligatory criteria 1‑Immediate skin sensitivity to *Aspergillus* or increased IgE against AF (>0.35 kUA/L)	2. Obligatory criteria 1‑Increased IgE against AF (>0.35 kUA/L) If this not available, Immediate skin sensitivity to AF may be considered
3. Immediate cutaneous reactivity to *Aspergillus* antigen	3. Elevated serum IgE and/or IgG against AF	3. Elevated serum IgE and/or IgG against AF	3. Obligatory criteria 2‑ Total serum IgE >1000 IU/ml (2400 ng/mL)	3. Obligatory criteria 2‑ Total serum IgE>1000 IU/ml (2400 ng/mL)
4. Precipitating antibodies against *Aspergillus* antigen	4. Total serum IgE conc. ( >417 kU/L or >1000 ng/mL)	4. Total serum IgE concentration > 417 kU/L (1000 ng/mL)		
5. Elevated total serum IgE (>1000 ng/mL)	5. Central bronchiectasis			
6. Chest X‑ray infiltrates (transient or fixed)				
7. Central bronchiectasis				
Secondary	Non-essential criteria		4. Other criteria: At least 2 of three	4. Other criteria: At least 2 of three
1. *Aspergillus fumigatus* in sputum (by culture or microscopy)	1. Chest X‑ray infiltrates	1. Chest X‑ray infiltrates	1. Radiographic findings consistent with ABPA*	1. Radiographic findings consistent with ABPA*
2. History of brown plugs in sputum	2. Serum precipitating antibodies to AF		2. Serum precipitating or IgG antibodies to AF	2. Serum IgG >27 mgA/L against AF
3. Late (Arthus) skin reaction to *Aspergillus* antigen			3. Increased total Eosinophils (>500) may be historical	3. Increased total Eosinophils (>500) may be historical
4. Increased total eosinophils (>500) may be historical				

ABPA should be considered in asthmatic patients exhibiting certain indicators. These include new or recent infiltrates on lung imaging, suggestive of pneumonia or tuberculosis, alongside clinical and laboratory evidence of asthma that remains uncontrolled despite appropriate treatment. Additionally, ABPA may be suspected in cases of unresolved pneumonia and bronchiectasis, regardless of asthma history. The potential differentials for ABPA encompass uncontrolled asthma, tuberculosis, pneumonia, foreign body inhalation, immotile cilia syndrome, pulmonary infiltrates with eosinophilia, cystic fibrosis without ABPA, and sarcoidosis [3].

Managing ABPA requires a dual strategy that involves both controlling the immune response to alleviate symptoms and prevent immune-related complications and reducing the organism's burden to minimize the host's exposure to the triggering stimulus [9]. The 2016 guidelines by the Infectious Diseases Society of America recommend oral corticosteroids to reduce inflammation in ABPA, with inhaled corticosteroids for asthma control in affected individuals [10]. For symptomatic patients, those with CF and low FEV1, or those with ABPA complicated by mucoid impaction, bronchiectasis, or chronic pulmonary aspergillosis, itraconazole is the first-line antifungal treatment [9].

The primary treatment approach for ABPA typically involves systemic glucocorticoids, supplemented by antifungal medications and anti-IgE therapy such as omalizumab. During exacerbations in stages 1 and 3, glucocorticoids are administered at a dose of 0.5-1mg/kg for 14 days, followed by a tapering regimen over three to six months, or longer if necessary, with alternate-day usage. Stage 2, characterized by remission, and stage 5, where fibrosis has developed, generally do not necessitate glucocorticoid therapy. Stage 4 signifies a scenario where attempts to taper glucocorticoids have failed, prompting the need for continued long-term therapy [11].

During exacerbations of ABPA, antifungal therapy using a 16-week course of itraconazole has shown efficacy in improving treatment response. This allows for a reduction in glucocorticoid dosage by 50%, along with a decrease in total serum IgE levels by 25% or more. In children, the recommended dose is 5 mg/kg/day administered in a single dose. If the calculated dose exceeds 200mg, it should be divided equally and given twice daily. However, caution is necessary, as a typical dosage regimen of 7mg/kg/day in children may lead to hepatotoxicity, necessitating close monitoring of liver function [11].

Antifungal therapy helps decrease the fungal burden, antigenic stimulus, and inflammatory response, thereby reducing the need for systemic corticosteroids [12]. Azole antifungals, like itraconazole, are usually administered at 200 mg twice daily for 16 weeks [13]. The combination of itraconazole and prednisolone has been shown to significantly lower the one-year exacerbation rate compared to the use of either drug alone [12].

## Conclusions

While ABPA in bronchial asthma is relatively uncommon, it should be considered in the list of potential causes of uncontrolled or difficult-to-treat asthma. Early identification before irreversible lung damage occurs is crucial for a favorable prognosis, emphasizing the importance of prompt treatment. Monitoring radiological findings and IgE levels is essential as clinical features alone may not reliably indicate ABPA progression or remission. Therefore, a comprehensive approach that includes vigilant observation of both imaging and immunological markers is necessary for effective management of ABPA in asthma patients.
